# A Case of a 23-Year-Old Male With Leber Hereditary Optic Neuropathy With a Rare Mutation

**DOI:** 10.7759/cureus.30198

**Published:** 2022-10-11

**Authors:** Priyal LNU, Vineet Sehgal, Lucky Bhalla Sehgal, Nihal Gulati, Saniya Kapila

**Affiliations:** 1 Neurology, Sehgal's Neuro & Child Care Centre, Amritsar, IND; 2 Neurology, Lady Hardinge Medical College, New Delhi, IND; 3 Neurology, Amandeep Medicity, Amritsar, IND; 4 Paediatrics, Sehgal's Neuro & Child Care Centre, Amritsar, IND; 5 General Practice, Navpreet Hospital, Amritsar, IND; 6 General Practice, Fortis Escorts Hospital, Amritsar, IND

**Keywords:** genetic eye diseases, sudden loss of vision, blindness without neurological deficit, mtdna disorder, mtdna mutation, rare genetic diseases, leber hereditary optic neuropathy

## Abstract

Mitochondrial DNA (mtDNA) is responsible for encoding 13 subunits of the respiratory chain. These subunits are crucial in providing reducing equivalents for the energy-intensive intracellular processes. Leber hereditary optic neuropathy (LHON) is a mitochondrial illness that causes carcinogenesis due to oxidative stress and painless loss of central vision as a result of selective degradation of retinal ganglion cells as well as their axons. We present a case of a 23-year-old male patient who was diagnosed with subacute LHON. The mutation in our patient was found in a less commonly mutated exon sequence of MT-NDL4, which codes for NADH (nicotinamide adenine dinucleotide hydrogen, reduced) dehydrogenase subunit 4L. The MT-ND4L exon is located immediately upstream of the MTD4 exon on the human mtDNA. The take-home message is to always perform a comprehensive mitochondrial genome analysis for identifying rare mutations when LHON is suspected.

## Introduction

Human mitochondrial DNA (mtDNA) has a mutation rate that is five to 10 times higher than nuclear DNA. It contains 16,569 base pairs that code for 22 mitochondrial transfer RNAs [1]. Four complexes make up the respiratory chain and are found inside the mitochondria. The mitochondrial respiratory chain consists of a total of 67 subunits. The mtDNA is responsible for encoding 13 of the subunits that are part of the respiratory chain (seven subunits of complex I, cytochrome b of complex III, three subunits of complex VI, and two subunits of adenosine triphosphate (ATP) synthase). These subunits are crucial in providing reducing equivalents for the energy-intensive intracellular processes.

The following are some recognized syndromes caused by dysfunctional mitochondrial mutations: mitochondrial encephalopathy, lactic acidosis, and stroke-like episodes (MELAS) syndrome; syndrome of myoclonic epilepsy with ragged red fibers (MERRF); neuropathy, ataxia, and retinitis pigmentosa (NARP) syndrome; Leber hereditary optic neuropathy (LHON); Pearson syndrome; Kearns-Sayre syndrome; maternally inherited diabetes and deafness (MIDD); and Leigh syndrome. One in every 50,000 people is thought to have LHON disease.

The mtDNA has 16 exonic sequences, and the MTD4 is one of the exons. MTD4 is one of the three most commonly mutated exon sequences in LHON disease. The mutation in our patient was found in a less commonly mutated sequence of MT-NDL4, which codes for NADH (nicotinamide adenine dinucleotide hydrogen, reduced) dehydrogenase subunit 4L. The MT-ND4L is a sequence located immediately upstream of the MTD4 gene on the human mtDNA.

## Case presentation

A 23-year-old man was admitted to our hospital with subacute onset sequential loss of vision over one month in both eyes. His vision loss began in the left eye and then progressed to the right eye one month later. In the past two months, he has had a steady decline in his vision to the point where he can now only see the perception of light. At the same time, he has been suffering from headaches on and off for the past three months. On general physical examination, he was afebrile, his heart rate was 98 per minute, he was normotensive, and the systemic examination was unremarkable. During the hospital course, neurology was called to the in-patient wards to evaluate what was thought to be optic neuropathy. He underwent an MRI of the brain to rule out compressive optic neuropathy. It included orbital cuts of the following sequences: spin echo (SE) T1-weighted, turbo spin echo (TSE) T2-weighted images, fluid-attenuated inversion recovery (FLAIR) sequence, and diffusion-weighted imaging (DWI) sequences in the axial plane. On a 1.5 Tesla scanner, he additionally went through a TSE T2-weighted sequence in the sagittal plane, a high-resolution fat-saturated (FS) TSE T2-weighted sequence, and a SE T1-weighted sequence in the coronal plane. On T2-weighted images thus acquired, the left optic nerve displayed a narrower breadth and faint hyperintensities, while on the right side, very similar but more subtle hyperintensities were seen.

**Figure 1:**
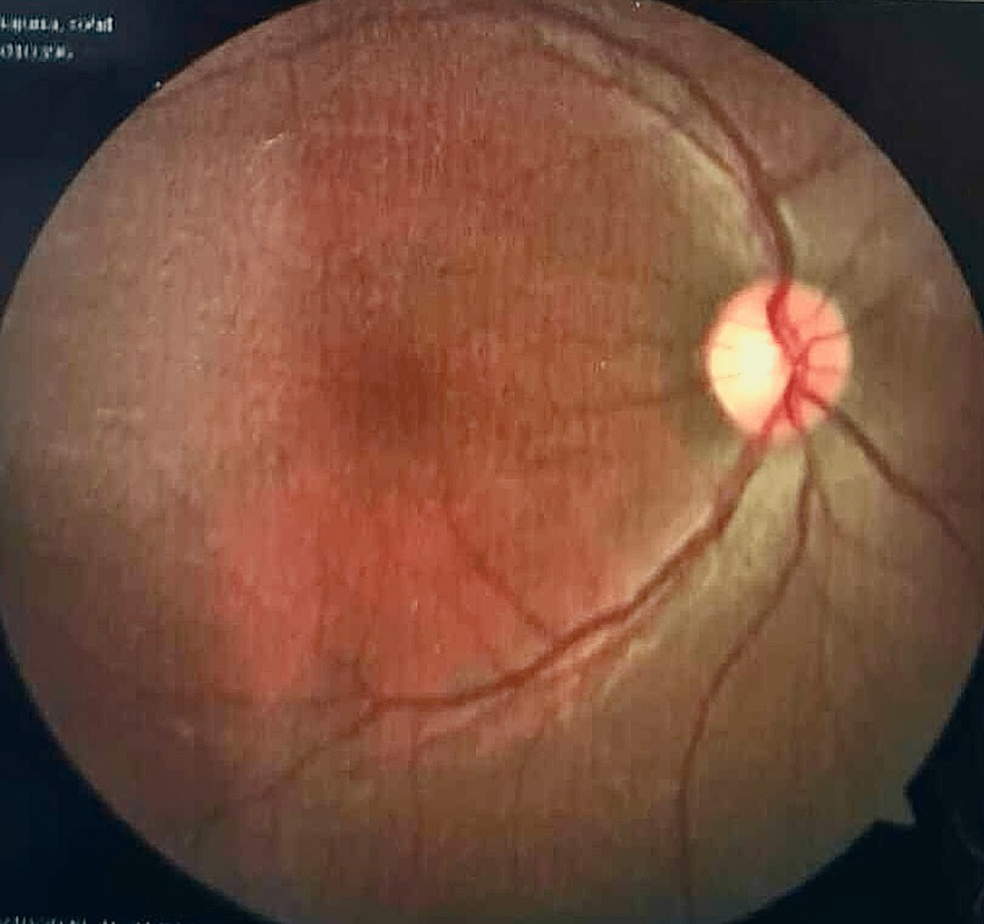
Right retina of the 23-year-old male patient with Leber hereditary optic neuropathy

**Figure 2:**
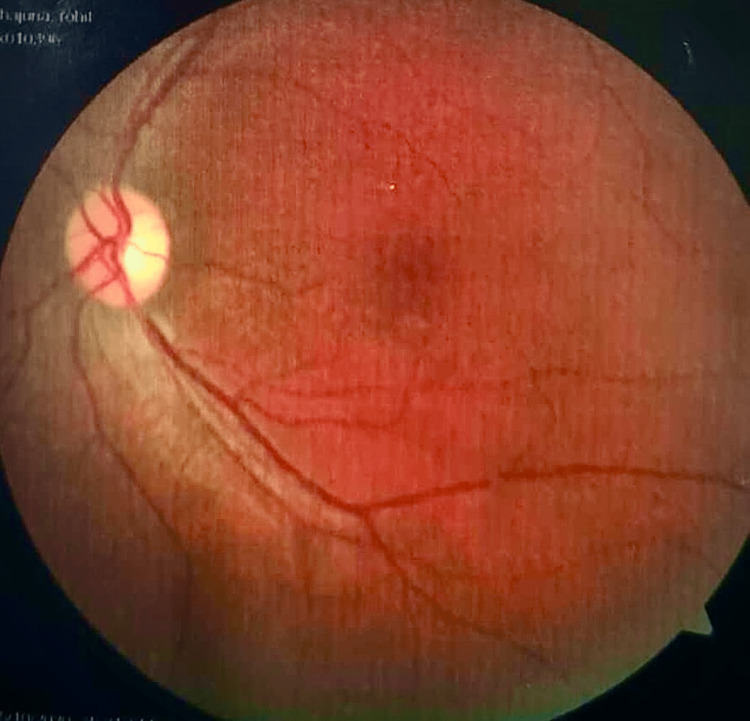
Left retina of the 23-year-old male patient with fulminant Leber hereditary optic neuropathy

These findings were suggestive of optic nerve atrophy. A contrast-enhanced MRI was performed to rule out orbital or cranial compressive mass. A CT angiography of the brain was normal and ruled out ischemic or stenotic lesions of the cranial vasculature. We evaluated the patient for the three most commonly occurring mutations in LHON syndrome patients, which are MTND1, MTND4, and MTND6, all of which came out negative. There was a strong suspicion of LHON. He underwent further evaluation of the whole mtDNA genome analysis for rare mitochondrial gene mutation, which showed a pathogenic homoplasmic (100% mutant mtDNA) missense mutation of the gene MT-ND4L that had resulted in the amino acid substitution of alanine for valine at codon 65 (p.Val65Ala). Indirect ophthalmoscopy showed optic nerve head pallor (Figures 1, 2). He was diagnosed with subacute LHON and clinical retrobulbar neuritis (Table 1).

**Table 1:** Results of the complete mitochondrial gene analysis of the patient showing the identification features of the variant sequence OMIM: Online Mendelian Inheritance in Man.

Gene (transcript)	Variant	Zygosity	Disease (MITOMAP and OMIM)	Classification
MT-ND4L (+) (ENST00000361335.1)	c.194T C (p.Val65Ala)	Homoplasmic	Leber optic atrophy	Pathogenic

He additionally underwent optical coherence tomography, visual evoked potentials, a comprehensive visual field examination, and the Ishihara card test. The results supported the diagnosis of LHON. His blood was examined for a complete blood count with differential counts, a complete metabolic panel, HIV, hepatitis A-E, venereal disease research laboratory (VDRL), angiotensin-converting enzyme (ACE) levels, antinuclear antibody (ANA), rheumatoid factor (RF), perinuclear antineutrophil cytoplasmic antibody (p-ANCA), cytoplasmic antineutrophil cytoplasmic antibody (c-ANCA), acute phase reactant proteins (C-reactive protein), vitamin B12 levels, folate levels, vitamin D levels, thiamine levels, serum ethyl alcohol levels, and blood heavy metal screening including lead levels. We did a CSF analysis for cell counts, glucose, proteins, culture, gram stain, India ink stain, VDRL, oligoclonal bands, and levels of IgG antibodies against myelin oligodendrocyte glycoprotein (MOG) and neuromyelitis optica (NMO). Thus, multiple sclerosis, NMO, toxic optic neuropathy, compressive optic neuropathy, and MOG antibody disease were ruled out by MRI, antibody assay, CSF, and blood analysis.

The patient was given dietary supplements such as vitamin D, calcium, and vitamin B12 to provide him with supportive care.

Variant interpretation and clinical correlation

The mitochondrial genome was completely covered. A homoplasmic missense variation in the MT-ND4L gene (chrM:10663T>C; depth: 2413x) that resulted in the amino acid substitution of alanine for valine at codon 65 (p.Val65Ala; ENST00000361335.1) was detected (Table 1). Mutations in the MT-ND4L gene have been reported to be associated with Leber optic atrophy.

The observed MT-ND4L (T10663C) variation has previously been reported as pathogenic in patients affected with Leber optic atrophy.

## Discussion

We presented a case of a 23-year-old male patient who was diagnosed with subacute LHON syndrome. The patient had a substitution mutation of alanine for valine at codon 65 of mtDNA, which is a rare mutation of two identical (non-polar and glycogenic) amino acids on the MT-ND4L exon, causing a subacute LHON disease. The MTD4 exon is more frequently known to be mutated in LHON illness than the MT-ND4L exon. LHON is a mitochondrial illness that causes bilateral painless loss of central vision as a result of selective degradation of retinal ganglion cells and their axons. The loss of P-type retinal ganglion cells, which project to the parvocellular layer of the lateral geniculate nucleus, may explain the clinical features of dyschromatopsia, central scotoma, and preservation of pupillary light response in LHON patients. This primarily parvocellular (red-green) pathway impairment conforms to the customary predilection of LHONs disease for affecting the papillomacular bundle and causing peripapillary microangiopathy, which is present from the onset of the disease but diminishes as the disease develops toward the final stages [2]. Loss of the retinal nerve fiber layer (RNFL) during the onset of LHON is characteristic of this condition. LHON is triggered by mutations in the primary mitochondrial DNA that exert an effect on the respiratory chain complexes. The subunits of the enzyme NADH-ubiquinone oxidoreductase (NADH dehydrogenase) are coded for by the exons ND1 through ND6. Seventeen mtDNA missense mutations have been proposed to contribute to LHON (MTND4 is one of the three primary mutations described in LHON, the other two mutations being MTND1 and MTND6 all code for subunit I of respiratory chain enzymes) [3,4]. A total of 90% of the LHON mutations seen the world over are one of these three. Approximately 20% of those with the MTND4 mutation see an improvement in their vision. The disorder has incomplete penetrance and demonstrates male predominance [3]. The progression of optic nerve involvement in each of the eyes can range from sudden and complete vision loss to progressive decline over two years, with a mean progression time of approximately 3.7 months. Both the final visual acuity and the amount of time needed for recovery are determined by the severity of the mutation as well as the type of mutation. In certain patients with LHON, cardiac conduction defects as well as neurological impairments have been described. LHON is known to be triggered by external factors such as trauma, cigarette smoking, alcohol consumption, and testosterone supplements [5,6]. The exact mechanism by which the LHON mutation is triggered by external factors and leads to the development of neuropathy or a phenotype similar to ocular atrophy is not completely understood. The cornerstones of treatment are supportive care and cessation of triggering substances such as medications and toxins such as alcohol and tobacco.

## Conclusions

We described the case of a 23-year-old male who was diagnosed with subacute LHON syndrome and had an uncommon mutation in the MT-ND4L exon of the mitochondrial DNA. The take-home message is to always perform a comprehensive mitochondrial genome analysis for identifying rare mutations whenever there is a strong suspicion of LHON and expand the epidemiological pool of rare mutations of the disease and establish a genotype-phenotype correlation.
